# A protocol for an updated and expanded systematic mixed studies review of fear of cancer recurrence in families and caregivers of adults diagnosed with cancer

**DOI:** 10.1186/s13643-018-0795-5

**Published:** 2018-08-31

**Authors:** Stuart Leske, Allan Ben Smith, Sylvie D. Lambert, Afaf Girgis

**Affiliations:** 1 Psycho-oncology Research Group, Centre for Oncology Education and Research Translation (CONCERT), Ingham Institute for Applied Medical Research, Liverpool, Australia; 2 South Western Sydney Clinical School, The University of New South Wales, Liverpool, Australia; 3 Ingram School of Nursing, McGill University, Montreal, Quebec Canada; 4 St. Mary’s Research Centre, Montreal, Quebec Canada

**Keywords:** Systematic review, Fear of cancer recurrence, Anxiety, Concern, Worry, Recurrence, Progression, Caregivers, Families

## Abstract

**Background:**

Fear of cancer recurrence (FCR) is reportedly common, persistent, associated with significant morbidity and often higher in cancer caregivers than cancer patients. This review will summarise empirical research on FCR to understand its prevalence, severity, correlates, course and impact in families and caregivers of adults diagnosed with cancer, and identify tested interventions that reduce its effects.

**Methods:**

This review will include peer-reviewed, empirical, qualitative and/or quantitative studies on fear, worry or concern of patients’ cancer returning or progressing among adult family members or caregivers of the cancer patient. It will exclude records reporting no original empirical research on FCR. We will search CINAHL, Embase, PubMed, PsycINFO, ProQuest Dissertations and Theses GLOBAL from 1997 onwards. Pairs of reviewers will conduct independent screening, data extraction and risk of bias assessment. Risk of bias will be assessed with the Cochrane Risk of Bias tool for randomised studies, the Risk of Bias Assessment tool for Nonrandomized Studies and the questions for qualitative studies in the mixed methods appraisal tool. We will conduct a narrative synthesis of quantitative studies and a thematic synthesis of qualitative studies.

**Discussion:**

This review will provide further clarity on the prevalence and severity of FCR in families and caregivers and differences by caregiver and care recipient demographic and medical characteristics. Any intervention studies located may indicate therapies or treatments that could reduce FCR in families and caregivers. Findings are expected to provide guidance for individuals and organisations working to manage FCR in families and caregivers of those with cancer.

**Systematic review registration:**

This protocol will be registered with PROSPERO after peer-review.

**Electronic supplementary material:**

The online version of this article (10.1186/s13643-018-0795-5) contains supplementary material, which is available to authorized users.

## Background

Fear of cancer recurrence (FCR) is fear, worry or concern about cancer returning or progressing [1]. FCR has been associated with existence and severity of physical symptoms and psychological distress [2], lower psychosocial wellbeing [3] and lower quality of life (QoL) [2, 3] in patients. It is one of the most frequently reported problems and unmet needs for both cancer survivors and their carers [2]. In patients, there is strong evidence of a positive association between FCR and distress, depression, anxiety and avoidance/intrusion and strong evidence of a negative association between FCR and QoL or functioning [2].

Caregivers have reported higher FCR than survivors’ FCR in all four studies that have made these comparisons [4–7]. In caregivers, FCR is negatively associated with QoL and positively associated with psychological distress [2]. FCR in family members is associated with family QoL 1 to 5 years after cancer treatment ends, contributing significantly to explaining variance in QoL when patient FCR did not [8]. FCR was one of the two most strongly associated variables with family caregivers’ QoL in a multivariable model, while cancer survivors’ FCR did not uniquely explain family caregivers’ QoL [6]. Associations between FCR and psychological distress are also higher over time in caregivers than the cancer patients they care for [4]. While substantially fewer studies assess FCR in caregivers compared to cancer patients, caregivers’ FCR remains important for short- and long-term adjustment to the illness, psychological and social functioning and overall QoL. There are also no known interventions to reduce FCR conducted with caregivers of cancer patients.

This review is needed as the previous systematic review [2] of FCR in adult cancer survivors, and their caregivers did not identify why caregivers’ FCR was significantly higher than survivors’ FCR in all four studies making these comparisons. New studies testing mediators and predictors may provide insights into why caregivers report more FCR. Including qualitative studies may provide a deeper, richer understanding of caregivers’ interpretations and experiences of FCR that complements and corroborates quantitative findings. We will include qualitative studies as caregiver samples are excluded [9] from the ongoing qualitative meta-synthesis of FCR [10].

In terms of interventions, strategies to assist caregivers with high FCR was identified as a priority area in the previous review [2] and a recently published proposal for a comprehensive FCR research program [11]. However, the ongoing review of psychological interventions for FCR in cancer patients [12] excludes caregiver samples [13], so we will include experimental studies in our review.

Lastly, we evaluated if the previous review [2] needed updating according to criteria for updating systematic reviews in a recently published consensus and checklist [14]. An updated review seems necessary because it still addresses a topical research question, there was significant interest in the original review, there have been advances in systematic review methodology since the previous review and there are new studies that might change or strengthen the original review’s findings. Including qualitative findings, new quantitative studies and using different review methodology may change or extend the earlier findings.

This review is expected to provide a better understanding of the prevalence and severity of FCR in caregivers and interventions that might reduce it. This will reduce uncertainty in clinical and support service practice and inform policy development in both settings. It will help clinicians to stay abreast of this literature and summarise research for cancer survivors, their families and caregivers. For researchers, this review will guide future research efforts to address identified gaps; develop, try, and evaluate new interventions and translate effective interventions into practice.

The primary audiences for the review are clinicians, researchers and support staff and services who support families and caregivers of people with cancer. This review has the perspective of clinical and support-service decision-making around managing FCR in families and caregivers.

This systematic review will summarise what is known from existing research on FCR in adult families and caregivers of adults with cancer. There are five main review questions, all concerning families and caregivers:

1. What is the prevalence and severity of FCR among family members and caregivers?
2. What variables correlate with family members’ and caregivers’ FCR (i.e. antecedents, mediators, moderators and outcomes)?
3. What is the course of family members’ and caregivers’ FCR over time?
4. How does FCR impact families’ and caregivers’ outcomes and adjustment to illness?
5. What impact do interventions have on reducing FCR and its outcomes?

## Methods

We developed this review methodology according to Cochrane Collaboration (CC) [15] and Centre for Reviews and Dissemination (CRD) guidance [16]. We report this protocol according to the preferred reporting items for systematic review and meta-analysis protocols (PRISMA-P) elaboration and explanation paper [17] (see Additional files 1 and 2; populated PRISMA-P and PRISMA Abstract checklists respectively).

### Eligibility criteria

Empirical studies presenting original data will be eligible. We will exclude commentaries, debates, editorials, guidelines, letters, opinions, responses and reviews, except when they report original and empirical data [18]. While not including them, we will examine conference abstracts to locate corresponding journal articles. Records must be peer-reviewed and published in writing, either internally or externally to authors’ institutions.

Studies will need to sample adult (i.e. aged ≥ 18) family members or adult caregivers of adults with cancer. We have excluded families and caregivers of children or adolescents with cancer, as they are a developmentally distinct group and the caregiver-care recipient relationship is likely to be very different.

Eligible studies will need to report results relating to FCR, excluding studies focusing solely on general or specific mental health symptoms or disorders unrelated to FCR. There are no restrictions on types of interventions, comparisons, contexts, settings, duration of studies or outcomes, so long as they co-occur with FCR assessment.

We will exclude studies in languages other than English or French, but list potentially relevant records in other languages in the appendix. Although the searches of the previous review occurred in December 2011 [2], the current review has wider scope by including qualitative studies and using additional search terms and strategies, so will overlap with the search period of the previous review by considering records published from 1997 onwards. This period is selected as the early seminal work on FCR which [19] was published in 1997.

This review will include both observational and experimental designs that collect quantitative or qualitative data or a mixture of both. There are no restrictions on study design, as we expect limited literature.

### Information sources and search strategy

Stuart Leske (SLe) will develop, pilot and will carry out the search strategy based on previous experience designing and revising search terms and strategies in four systematic reviews and CC [15] and CRD [16] guidance. SLe will refine the search strategies before piloting with all other authors who are content experts in FCR and an academic services librarian for medicine.

The review will search for records indexed in CINAHL, Embase, PubMed and PsycINFO. Table 1 lists the keywords that we will use to search these databases. Keywords refer to information about the population (e.g. famil* and caregiver*), combined with FCR terms (e.g. fear* and worr*), recurrence terms (e.g. recur* and reoccur*) and cancer terms (e.g. cancer* and neoplasm*).

**Table 1:** Complete search strategy for all electronic bibliographic databases

	Population terms (search 1) AND	FCR terms (search 2) AND	Recurrence terms (search 3) AND	Cancer terms (search 4)
Terms that searches below are intended to capture	Family, families, caregiver, caregivers, caregiver, caregivers, caregiving, spouse, spouses, spousal, dyad, dyads, dyadic, partner, partners, couple, couples, coupled	Afraid, anxiety, anxieties, fear, fears, fearing, feared, worry, worrying, worries, worried	Recur, recurs, recurrence, recurring, recurred, relapse, relapsed, relapsing, progress, progression, progressed, progressing, coming back, reoccur, reoccurs, reoccurrence, reoccurring, reoccurred, returned, returning, return, returns.	Cancer, cancers, umor, tumors, tumour, tumours
Database				
CINAHL via EBSCOhost	Title/abstract/subject searches: Famil* OR caregiver* or caregiver* or spous* or dyad* or partner* or couple* OR CINAHL Headings: (MH ‘Family+’) OR (MH ‘Caregivers’)	Title/abstract/subject searches: afraid or anxiet* or fear* or worr* OR CINAHL Headings: (MH ‘Fear+’)	Title/abstract/subject searches: coming back or progress* or recur* or relaps* or reoccur* or return* or spread* OR CINAHL Headings: (MH ‘Disease Progression’) OR (MH ‘Recurrence’)	Title/abstract/subject searches: Cancer* or tumor* or tumour* OR CINAHL Headings: (MH ‘Neoplasms+’)
EMBASE via Ovid	Title/abstract/keyword searches: Famil* OR caregiver* or caregiver* or spous* or dyad* or partner* or couple* OR Emtree: exp. family/ or exp. caregiver/ or exp. caregiver burden/ or exp. caregiver strain index/or exp. caregiver support/or exp. dyad/	Title/abstract/keyword searches: afraid or anxiet* or fear* or worr* OR Emtree: exp. fear/	Title/abstract/keyword searches: coming back or progress* or recur* or relaps* or reoccur* or return* or spread* OR Emtree: exp. cancer recurrence/ or exp. cancer regression/ or exp. relapse/ or exp. recurrent disease/ or exp. tumor recurrence/ or exp. tumor regression/or exp. progression free survival/	Title/abstract/keyword searches: Cancer* or tumor* or tumour* OR Emtree: exp. neoplasm/
ProQuest Dissertations and Theses – GLOBAL via ProQuest	Title/abstract/index term (keyword) searches: Famil* OR caregiver* or caregiver* or spous* or dyad* or partner* or couple* OR Subject searches: Exact(‘families & family life’ OR ‘caregivers’ OR ‘single family’ OR ‘partners’ OR ‘individual & family studies’ OR ‘couples’ OR ‘domestic partners’)	Title/abstract/index term (keyword) searches: afraid or anxiet* or fear* or worr* OR Subject searches: Exact(‘worry’ OR ‘fear & phobias’)	Title/abstract/index term (keyword) searches: coming back or progress* or recur* or relaps* or reoccur* or return* or spread* OR Subject searches: Exact(‘progress’)	Title/abstract/index term (keyword) searches: Cancer* or tumor* or tumour* OR Subject searches: Exact(‘skin cancer’ OR ‘colorectal cancer’ OR ‘esophageal cancer’ OR ‘ovarian cancer’ OR ‘uterine cancer’ OR ‘fear & phobias’ OR ‘breast cancer’ OR ‘thyroid cancer’ OR ‘brain cancer’ OR ‘cervical cancer’ OR ‘genital cancers’ OR ‘kidney cancer’ OR ‘lung cancer’ OR ‘testicular cancer’ OR ‘pancreatic cancer’ OR ‘prostate cancer’ OR ‘bone cancer’ OR ‘endometrial cancer’ OR ‘progress’ OR ‘cancer’ OR ‘liver cancer’ OR ‘oral cancer’ OR ‘head & neck cancer’
PsycINFO via Ovid	Title/abstract/key concepts searches: Famil* OR caregiver* or caregiver* or spous* or dyad* or partner* or couple* OR Subjects: exp. FAMILY/ or exp. family members/ or exp. Caregivers/ or exp. Caregiver Burden/or exp. couples/	Title/abstract/key concepts searches: afraid or anxiet* or fear* or worr* OR Subjects: exp. fear/or exp. anxiety/	Title/abstract/key concepts searches: coming back or progress* or recur* or relaps* or reoccur* or return* or spread* OR Subjects: exp. ‘relapse (disorders)’/	Title/abstract/key concepts searches: Cancer* or tumor* or tumour* OR Subjects: exp. neoplasms/
PubMed via National Library of Medicine	Title/abstract searches: Famil* OR caregiver* or caregiver* or spous* or dyad* or partner* or couple* OR MeSH terms: ‘Family’[Mesh] OR ‘Caregivers’[Mesh]	Title/abstract searches: afraid or anxi* or fear* or worr* OR MeSH terms: ‘Fear’[Mesh] OR ‘Anxiety’[Mesh]	Title/abstract searches: coming back or progress* or recur* or relaps* or reoccur* or return* or spread* OR MeSH terms: ‘Recurrence’[Mesh] OR ‘Disease Progression’[Mesh] OR ‘Disease-Free Survival’[Mesh]	Title/abstract searches: Cancer* or tumor* or tumour* OR MeSH terms: ‘Neoplasms’[Mesh]

One author will search for grey literature (see Table 2) through Google (restricted to pdfs and word documents), Open Grey and WorldCat. One author will retrieve clinical trial protocols from 17 registries on the World Health Organization (WHO) International Clinical Trials Registry Platform. We will review conference abstracts from six major psycho-oncology conferences to cross-check for studies not have been located in other searches.

**Table 2:** Sources searched to retrieve grey literature

Type of grey literature	Organising body and conference name	Search strategy and outlet	Years
Conference abstracts	International Psycho-Oncology Society World Cancer Congress	Review supplementary issues of *Psycho-Oncology* (journal)	2008 onwards
	American Psychosocial Oncology Society Annual Conference	Review supplementary issues of *Psycho-Oncology* (journal)	2009 onwards
	British Psychosocial Oncology Society Annual Conference	Review supplementary issues of *Psycho-Oncology* (journal)	1997, 2010, 2012–2017
	Clinical Oncology Society of Australia Annual Scientific Meeting	Review supplementary issues of Asia-Pacific Journal of Clinical Oncology (journal)	2005 onwards
	Psycho-Social Oncology New Zealand conferences	Review organisation website: http://ponz.org.nz/index.php?page=conferences	2008 onwards
	Multinational Association of Supportive Care in Cancer Annual Meetings	Review supplementary issues of *Supportive Care in Cancer* (journal)	2012 onwards
Clinical trials	Australian New Zealand Clinical Trials Registry (ANZCTR)	Keyword search on WHO search portal: http://apps.who.int/trialsearch/	1997 onwards
	Brazilian Clinical Trials Registry (ReBec)		
	Chinese Clinical Trial Register (ChiCTR)		
	Clinical Research Information Service (CRiS), Republic of Korea		
	ClinicalTrials.gov		
	Clinical Trials Registry - India (CTRI)		
	Cuban Public Registry of Clinical Trials (RPCEC)		
	EU Clinical Trials Register (EU-CTR)		
	German Clinical Trials Register (DRKS)		
	Iranian Registry of Clinical Trials (IRCT)		
	ISRCTN.org		
	Japan Primary Registries Network (JPRN)		
	Pan African Clinical Trial Registry (PACTR)		
	Peruvian Clinical Trials Registry (REPEC)		
	Sri Lanka Clinical Trials Registry (SLCTR)		
	Thai Clinical Trials Register (TCTR)		
	The Netherlands National Trial Register (NTR)		
European grey literature e.g., technical or research reports, doctoral dissertations, some conference papers, some official publications	Open Grey - System for Information on Grey Literature in Europe	Keyword search on website: http://www.opengrey.eu/	1997 onwards
All grey literature types	Google search	Keyword search on website, restricted to PDF documents or .org and/or.gov domains: https://www.google.com.au/	1997 onwards
All grey literature types	WorldCat	Keyword, subject and title searches on advanced search page: http://www.worldcat.org/advancedsearch	1997 onwards
All types of grey literature in health services research and selected urban health topics	The Grey Literature Report	Keyword and MeSH term searching on website: http://greylit.org/	1997–2016
All grey literature types	The Grey Literature Network Service – GreyNet International	Keyword search on website: http://greyguide.isti.cnr.it/	1997 onwards

One author will examine the reference lists of included studies or relevant reviews identified from the searches. Authors of included studies will be emailed to enquire about unpublished research. After screening concludes, a bibliography of included records will be circulated to the systematic review team and the Fear of Cancer Recurrence Special Interest Group of the International Psycho-Oncology Society for review to identify any studies that have been missed. This search has time and financial constraints, resource constraints as librarians have limited scope to be involved, and language constraints.

### Study records

#### Data management

We will export study records from the databases into the Systematic Review Assistant-Deduplication Module (SRA-DM) [20] developed by the Centre for Research in Evidence-Based Practice at Bond University. The SRA-DM had superior sensitivity (84%) and specificity (100%) to EndNote’s deduplication function (sensitivity 51%, specificity 99.8%) in a recent deduplication of 1988 citations [20].

We will then export records to Covidence systematic review software [21] to enable screening of titles and abstracts and then full-text records by pairs of reviewers. The research team will develop and test screening questions based on eligibility criteria in Covidence, then conduct a calibration exercise on a random sample of 20 records to pilot and refine the questions before formal screening begins.

Full-text records will be retrieved and uploaded to the associated citations in Covidence. To avoid double-counting multiple reports from the same study as multiple studies, data extractors will compare author names, measurement of FCR and other outcomes, sample sizes and study settings. Where inconsistencies in multiple reports from the same study are apparent and unresolved among the research team, a review author will contact study authors to clarify information. Allan ‘Ben’ Smith (ABS) will have overall responsibility for data management.

#### Selection process

Pairs of reviewers will independently screen titles and abstracts and then full-text records of records reaching the second stage of screening. We will be over-inclusive at the first stage and include all reports appearing to meet the inclusion criteria or those where eligibility is uncertain. We will seek further information from study authors if necessary to confirm eligibility at the second stage of screening. Reasons for exclusion will be recorded only at the second stage of screening. Pairs will resolve discrepancies via consensus and discuss unresolved discrepancies with a third researcher. Reviewers will not be blinded to the study authors, institutions or journals of the records they screen. We will calculate inter-rater reliability (Cohen’s kappa) for each screening stage and report this in the full review.

#### Data collection process

We will pilot a data extraction form with detailed instructions in Microsoft Word on a subsample of included papers to assess the efficiency of the form and the accuracy and consistency of extractors. The draft forms for experimental and observational studies are attached as Additional files 3 and 4 respectively.

The form is based on the Cochrane Handbook’s *Checklist of items to consider in data collection or data extraction* (Table 7.3.a in Version 5.1.0 of the Cochrane Handbook) and the *Example information requirements for data extraction*, Box 1.4, in the Centre for Reviews and Dissemination’s guidance for undertaking reviews in healthcare [16]. Data items added or removed after protocol development and review initiation will be documented in amendments and the completed review per the process described in the discussion section.

Reasonable assumptions made when extracting data (e.g. ‘adults’ mostly refers to individuals aged ≥ 18) will be mentioned in the full review. One reviewer will contact study authors via email (with a maximum of three email attempts) to obtain and confirm missing or unclear information, if needed. We will note this data as sought and/or received in the data extraction tables. Where authors are uncontactable and apparent discrepancies between reports exist, we will use the report with better methodological quality.

#### Data items

For source identification, we will assign study, report and extractor identification details to each record. We will also include the type of publication (e.g. journal article and conference abstract) and the country of origin.

For methodology, we will note theories underpinning studies. For methods, we will include the study design, assessment/follow-up details (length, number and time points) where applicable and study setting (e.g. hospital and supportive care institution). In all studies, we will note the sampling strategy and data collection method (e.g. survey and interview).

For participant characteristics, we will record the sample subgroups as defined by study authors, total number of families and/or caregivers, their relationship to the patient and caregiving status (e.g. primary, secondary, sole and dual), inclusion and exclusion criteria in studies if any, and their age, sex, co-morbidities, socio-demographics and ethnicity. We will also note the type and stage of the care recipient’s cancer.

For intervention characteristics, we will record the total number of intervention groups, the specific type and sub-type of intervention, intervention components, any reported integrity or fidelity of the intervention, co-interventions, the intervention setting and delivery agent. We will also record the dose, method of administration, duration and number and frequency of intervention sessions, noting if the detail provided is sufficient for replication.

For comparator characteristics in observational studies, we will describe the characteristics serving as a reference point in a manner consistent with the ‘participant section’ above. In experimental studies, we will record the control condition characteristics according to the intervention characteristics described above.

For outcome characteristics, we will record the measure used, means, standard deviations, range, effect sizes and clinically significant cut-offs of FCR and the relationship of all secondary outcomes to FCR using statistics appropriate to the hypothesised relationship.

For experimental results, we will describe the type of analysis used (e.g. intention-to-treat and per protocol); the number of participants eligible, allocated to each intervention group, and analysed; the sample size, missing participants and summary data for each intervention group and the reported estimates of effect, confidence limits and significance values. For qualitative studies, we will report on the type of data analysis (e.g. grounded theory and thematic analysis) and analytic strategy used (e.g. constant comparative method). In a miscellaneous section, we will note funding sources and conflicts of interest.

### Outcomes and prioritisation

FCR will be prioritised as the primary outcome. Secondary outcomes include all variables assessed alongside FCR in empirical studies that have been modelled as either predictor, associated (i.e. correlated) or outcome variables relative to FCR. We are interested in all outcomes to identify if intervention is possible before or after FCR development to prevent it from occurring or reduce its effects. These secondary outcomes may be physical or psychological symptoms or conditions, health care factors, coping strategies, QoL-related or health behaviours.

### Risk of bias of individual studies

Two reviewers will independently assess risk of bias at the study (e.g. adequate sequence generation) and outcome (e.g. blinding) level. Reviewers will not be blinded to the authors, institutions or journals of included studies. A pre-designed Microsoft Word template will be used to conduct these assessments. Appraising reviewers will resolve disagreements about risk of bias by discussion, involving another reviewer where necessary. We will calculate and report inter-rater agreement (Cohen’s kappa) in the full review.

We will assess risk of bias in RCTs with the Cochrane Risk of Bias tool [22] and in non-randomised studies with the Risk of Bias Assessment Tool for Nonrandomized Studies (RoBANS) [23]. Both tools have six domains. Readers are referred to these documents for the names and definitions of the constructs assessed.

Assessments of overall risk of bias will consider empirical evidence of bias and its likely direction and magnitude, and assessments will be summarised for individual outcomes within a study (across domains), in accordance with recommendations from section 8.7 of the Cochrane Handbook [24]. Overall assessments will be a ‘high risk’ of bias, ‘low risk’ of bias or ‘unclear risk’ of bias. Individual studies will be ‘high risk’ overall if there is a high risk of bias for one or more key domains [24]. Low risk of bias will be assigned when the study has a low risk of bias for all key domains, and unclear risk of bias will be used when risk of bias is unclear for one or more key domains [24]. To minimise unclear risk, we will contact study authors for more information.

We will draw attention to the methodological quality of studies during stage two of the narrative synthesis, where we will explore reasons for heterogeneous findings within and across studies. We will retain all studies in narrative and thematic syntheses but cite any external empirical evidence of bias for the domains in question and discuss the potential effect this has on the direction and magnitude of estimates (quantitative studies) or findings (qualitative studies).

We chose the Cochrane Risk of Bias tool, as it assesses trial conduct and not reporting; it was developed based on wide consultation, empirical and theoretical evidence and it places an emphasis on transparency [25]. It is being evaluated and updated, which will likely consider new empirical evidence and the existing weaknesses identified with the tool [22]. More empirical evidence of bias and discussion about bias it does not cover (e.g. sponsorship bias) has recently (June 2017) been added to the Cochrane Handbook.

We chose RoBANS, as it is suitable for all study designs except RCTs, and it has moderate reliability, feasibility and validity [23]. Furthermore, it is designed to be consistent in length and focus with the Cochrane Risk of Bias tool, enabling consistent evaluation of bias across diverse study designs.

For qualitative or mixed-methods studies, we will use the relevant sections of the mixed-methods appraisal tool (MMAT) [26]. The current (2011) version of the MMAT includes two screening questions for all study types, four questions for qualitative studies and three questions for mixed-methods studies. These domains have been content-validated by developing the items from the literature and through consultations and workshops with experts [26–28]. The MMAT is feasible (i.e. short to administer [27, 29]). Items assessing qualitative studies have lower kappa [27, 29] so we will pilot the appraisal form on a subset of studies to ensure appraisers have a common understanding of these criteria before starting the quality assessment [29].

### Data synthesis

Clinical, methodological and statistical heterogeneity will likely preclude meta-analysis. We will aggregate data and use a sequential explanatory synthesis. In this synthesis, the quantitative synthesis precedes and informs the qualitative synthesis, and the qualitative synthesis helps to explain some findings from the quantitative synthesis [30]. The quantitative synthesis will be narrative (descriptive), informed by guidance from the United Kingdom’s Economic and Social Research Council’s Methods Programme [31–34]. We will use this guidance to develop a preliminary synthesis of the results, explore relationships within and between studies and assess the robustness of the synthesis. Developing a preliminary synthesis will involve organising findings from included studies to describe patterns across the studies in the prevalence and severity of FCR or relationship of FCR to other variables. We will explore relationships within and between studies that might explain any heterogeneity in their findings, drawing on the risk of bias assessments. Sources of potential heterogeneity may arise from study designs, populations, interventions, comparators, outcomes, contexts and/or settings. Assessing the robustness of the synthesis will involve appraising the quality and relevance of evidence from high quality studies (i.e. those without high risk of bias) for each of our research questions using the weight of evidence framework described by Gough [35, 36]. This final stage will assess the coherence and integrity of research evidence (weight of evidence A), the appropriateness of individual study designs for answering each research question (weight of evidence B), the relevance of the focus of the evidence for each research question (weight of evidence C) and an overall assessment of the extent to which an individual study contributes towards answering a research question (weight of evidence D) [35, 36]. These justifications will be documented and thus transparent in the full review.

The qualitative synthesis will be a thematic analysis, informed by guidance from Thomas and Harden [37]. This will be a three-stage process, starting with line-by-line coding of the meaning and content of findings from primary studies, then organising coding hierarchically in a tree structure into related areas to develop ‘descriptive’ themes and lastly, creating ‘analytical’ themes [37]. Pairs of reviewers will independently review the findings of included studies to develop coding structures. Similar to Thomas and Harden, we define findings as the complete text classified as ‘results’ or ‘findings’ in reports of studies, including in the abstracts. We will create analytical themes that make inferences and go beyond the original data by using the descriptive themes to answer the research questions we initially posed. Pairs of reviewers will independently generate analytic themes and then meet to discuss any inconsistencies. We will make inferences informed by the findings of the quantitative synthesis, but the qualitative synthesis may produce additional insights that explain the quantitative findings or are distinct from them. We will structure both syntheses to answer the five review questions we posed, giving equal priority to discussing research with families and/or caregivers. The text will seek to explore important differences between and within studies based on study designs and information from the data extraction and quality appraisal. In each section, we will report quantitative then qualitative studies. Tables will be used to summarise the characteristics of studies, findings and outcomes, and the text will describe these findings in aggregate form and note important differences.

### Updated methods

The review methods differ from and expand upon the review it partially updates [2] in several ways. We will examine FCR in families and caregivers only, which was a component of the previous review.

We will not search the Allied and Complementary Medicine Database or Web of Science, but will instead search Embase to retrieve records of studies on mainstream medicines not captured by PubMed. We will also include any records subjected to any peer-reviewed process, such as theses and grey literature. We will search multiple databases and registries to capture clinical trials, student theses and dissertations and other grey literature. The search terms in this review will be different, as there are family- and caregiver-specific search terms, five more keywords, ten more wildcard symbols and indexing terms used in all databases. This review will include only English- and French-language records, whereas the previous review also included records published in German or Turkish [2]. However, all 130 records included in the previous review had English titles, indicating that they were likely all English-language records.

During study selection, pairs of reviewers will independently examine all records for inclusion, meeting to resolve discrepancies and involving a third reviewer where necessary. For risk of bias, we will assess and comment on the methodological quality of studies using domain-based evaluation tools appropriate for the study design.

## Discussion

### Procedure for protocol amendments

Following the Agency for Healthcare Research and Quality’s Effective Health Care Program process for handling protocol amendments [17], we will record the date of each amendment, the location of the change in the protocol, a description of the language in the original protocol, a description of the change in the protocol and a rationale that specifies why the change will improve the report, why the change does not introduce bias (if necessary) and the expected outcomes of the change. Amendments will be made in PROSPERO and the full review, but not as an erratum to the protocol paper. AG will have final responsibility for approving the amendments and ABS will date, document and implement amendments.

### Limitations

We acknowledge that there are numerous measures of FCR available (for a review see [38]), and no clear consensus about which measure is best, particularly for identifying likely instances of clinically significant FCR. This makes synthesising FCR results difficult, particularly those relating to the prevalence of FCR. Due to the heterogeneity in FCR measures, we will undertake a narrative synthesis, rather than meta-analysis of quantitative results.

### Dissemination plans

We expect to present findings orally and in writing to stakeholders in the care and support of cancer survivors and their families and caregivers. This may include survivors, families, caregivers, researchers and clinicians at our research group, institute, university, state and federal government cancer departments and non-government organisations. We intend to publish the findings in a peer-reviewed journal (open-access if possible) and upload the manuscript accepted for publication to our university’s institutional repository.

## Additional files

Additional file 1: PRISMA-P 2015 Checklist. (PDF 233 kb)

Additional file 2: The PRISMA for Abstracts Checklist. (PDF 189 kb)

Additional file 3: Data Extraction Template: Experimental studies. (PDF 69 kb)

Additional file 4: Data Extraction Template: Observational Studies. (PDF 57 kb)

## Abbreviations

CC: Cochrane Collaboration
CINAHL: Cumulative Index to Nursing and Allied Health Literature
CONCERT: Centre for Oncology Education and Research Translation
CRD: Centre for Reviews and Dissemination
Embase: Excerpta Medica dataBASE
FCR: Fear of cancer recurrence
FE: Fixed effects
GRADE: Grading of Recommendations Assessment, Development and Evaluation
NSW: New South Wales
PRISMA-P: Preferred reporting items for systematic review and meta-analysis protocols
PROSPERO: International prospective register of systematic reviews
QoL: Quality of Life
RCTs: Randomised controlled trials
RoBANS: Risk of Bias Assessment tool for Nonrandomized Studies
RR: Risk ratio
SRA-DM: Systematic Review Assistant-Deduplication Module
WHO: World Health Organization
