# Supplementary material for: A protocol for an updated and expanded systematic mixed studies review of fear of cancer recurrence in families and caregivers of adults diagnosed with cancer
Source: Syst Rev. 2018 Aug 31;7:134. doi: 10.1186/s13643-018-0795-5 (PMC6119342; doi:10.1186/s13643-018-0795-5)
Supplement: Supplementary file 2 — The PRISMA for Abstracts Checklist. (PDF 189 kb) [file 13643_2018_795_MOESM2_ESM.pdf]

The PRISMA for Abstracts Checklist

TITLE	CHECKLIST ITEM	REPORTED ON PAGE #
1. Title:	Identify the report as a systematic review, meta-analysis, or both.	1
**BACKGROUND**		
2. Objectives:	The research question including components such as participants, interventions, comparators, and outcomes.	2
**METHODS**		
3. Eligibility criteria:	Study and report characteristics used as criteria for inclusion.	2
4. Information sources:	Key databases searched and search dates.	2
5. Risk of bias:	Methods of assessing risk of bias.	2
**RESULTS**		
6. Included studies:	Number and type of included studies and participants and relevant characteristics of studies.	N/A
7. Synthesis of results:	Results for main outcomes (benefits and harms), preferably indicating the number of studies and participants for each. If meta-analysis was done, include summary measures and confidence intervals.	N/A
8. Description of the effect:	Direction of the effect (i.e. which group is favoured) and size of the effect in terms meaningful to clinicians and patients.	N/A
**DISCUSSION**		
9. Strengths and Limitations of evidence:	Brief summary of strengths and limitations of evidence (e.g. inconsistency, imprecision, indirectness, or risk of bias, other supporting or conflicting evidence)	N/A
10. Interpretation:	General interpretation of the results and important implications	N/A
**OTHER**		
11. Funding:	Primary source of funding for the review.	20-21
12. Registration:	Registration number and registry name.	2
