# Supplementary material for: A protocol for an updated and expanded systematic mixed studies review of fear of cancer recurrence in families and caregivers of adults diagnosed with cancer
Source: Syst Rev. 2018 Aug 31;7:134. doi: 10.1186/s13643-018-0795-5 (PMC6119342; doi:10.1186/s13643-018-0795-5)
Supplement: Supplementary file 3 — Data Extraction Template: Experimental studies. (PDF 69 kb) [file 13643_2018_795_MOESM3_ESM.pdf]

Additional File 3. Data Extraction Template: Experimental studies

	Study ID	
	Report ID	
	Extractor	
**Source details**	Author	
	Year	
	Title	
	Type of publication	
	Country of origin	
**Methodology**	Theoretical framework	
	Study design	
	Assessment/follow-up: Length	
	Assessment/follow-up: Number	
	Assessment/follow-up: Time Points	
	Study setting	
	Sampling strategy	
	Data collection method	
**Participant characteristics** (Same for comparator groups)	Sample subgroups	
	Total number of families/caregivers	
	Relationship to patient	
	Caregiving status (e.g., primary, secondary, sole, dual)	
	Inclusion criteria	
	Exclusion criteria	
	Age	
	Sex	
	Co-morbidities	
	Marital Status	
	Education	
	Employment	
	Ethnicity	
	Type of cancer of care recipient	
	Stage of cancer of care recipient	
**Intervention**	Total number of groups	
	Type/sub-type of intervention	
	Intervention components	
	Reported integrity or fidelity	
	Co-intervention	
	Intervention setting	
	Delivery agent	
	Dose	
	Method of administration	
	Duration	
	Number of sessions	
	Frequency of sessions	
	Details provided sufficient for replication?	
**Control**	Total number of groups	
	Type/sub-type of control	
	Control components	
	Reported integrity or fidelity	
	Co-control	

	Control setting	
	Delivery agent	
	Dose	
	Method of administration	
	Duration	
	Number of sessions	
	Frequency of sessions	
	Details provided sufficient for replication?	
**Outcomes**	FCR measure used	
	Mean, SD, Range, Effect Size, Clinically Significant Cut-offs (FCR)	
	Relationship of all secondary outcomes to FCR	
	Type of analysis used (e.g., intention to treat, per protocol)	
	Number of participants eligible (Intervention)	
	Number of participants allocated (Intervention)	
	Number of participants analysed (Intervention)	
	Sample size (Intervention)	
	Missing participants (Intervention)	
	Summary data (Intervention)	
	Number of participants eligible (Control)	
	Number of participants allocated (Control)	
	Number of participants analysed (Control)	
	Sample size (Control)	
	Missing participants (Control)	
	Summary data	
	Differences in FCR outcomes between Control and Intervention (Reported estimates of effect, confidence limits, and significance values)	
	FCR themes/sub-themes (Qualitative)	
	Type of data analysis (Qualitative)	
	Analytic strategy used (Qualitative)	
**Miscellaneous**	Funding sources	
	Conflicts of interest	
