# Supplementary material for: A protocol for an updated and expanded systematic mixed studies review of fear of cancer recurrence in families and caregivers of adults diagnosed with cancer
Source: Syst Rev. 2018 Aug 31;7:134. doi: 10.1186/s13643-018-0795-5 (PMC6119342; doi:10.1186/s13643-018-0795-5)
Supplement: Supplementary file 4 — Data Extraction Template: Observational Studies. (PDF 57 kb) [file 13643_2018_795_MOESM4_ESM.pdf]

Additional File 4. Data Extraction Template: Observational Studies

	Study ID	
	Report ID	
	Extractor	
**Source details**	Author	
	Year	
	Title	
	Type of publication	
	Country of origin	
**Methodology**	Theoretical framework	
	Study design	
	Assessment/follow-up: Length	
	Assessment/follow-up: Number	
	Assessment/follow-up: Time Points	
	Study setting	
	Sampling strategy	
	Data collection method	
**Participant characteristics** (Same for comparators)	Sample subgroups	
	Total number of families/caregivers	
	Relationship to patient	
	Caregiving status (e.g., primary, secondary, sole, dual)	
	Length of relationship	
	Inclusion criteria	
	Exclusion criteria	
	Age	
	Sex	
	Co-morbidities	
	Marital Status	
	Education	
	Employment	
	Ethnicity	
	Type of cancer of care recipient	
	Stage of cancer of care recipient	
**Outcomes**	FCR Measure	
	Mean, SD, Range, Effect Size, Clinically Significant Cut-offs (FCR)	
	Relationship of all secondary outcomes to FCR	
	FCR themes/sub-themes (Qualitative)	
	Type of data analysis (Qualitative)	
	Analytic strategy used (Qualitative)	
**Miscellaneous**	Funding sources	
	Conflicts of interest	
